# Evaluation of the donor site defect after harvesting the medial third of the patellar tendon in ACL reconstruction: An MRI study

**DOI:** 10.1002/jeo2.70571

**Published:** 2025-11-28

**Authors:** Jim Georgoulis, Olga Savvidou, Paraskevi Kosta, Kostas Patras, Maria Argyropoulou, Panayiotis Papagelopoulos, Anastasios Georgoulis

**Affiliations:** ^1^ 1st Department of Orthopaedic Surgery National and Kapodistrian University of Athens Athens Greece; ^2^ Department of Orthopedics, Orthopaedic Sports Medicine Center of Ioannina University of Ioannina Ioannina Greece; ^3^ Radiology Department, Medical School University Hospital of Ioannina Ioannina Greece

**Keywords:** anterior cruciate ligament, healing rate 5, medial third graft 3, MRI 4, patellar tendon 2

## Abstract

**Purpose:**

Previous studies have reported magnetic resonance imaging (MRI) changes in the donor‐site defect size following anterior cruciate ligament reconstruction (ACLR) using the middle third of the patellar tendon (mid‐third patellar tendon ACLR); however, no corresponding reports exist for ACLR using the medial third of the patellar tendon (medial‐third patellar tendon ACLR). This study aimed to assess the changes in donor‐site defect size postoperatively in medial‐third ACLR patients and hypothesized a clinically relevant MRI change within the first year.

**Methods:**

Twenty‐two consecutive patients underwent MRI examinations to measure the donor‐site defect size (‘gap’ signal) and laterally remaining tendon size (‘normal tendon’ signal) at two time points: 14 (±5) days and 7.9 (±2.0) months postoperatively. The primary outcome was the post‐pre difference of the “gap” signal. Wilcoxon signed rank test assessed the post–pre difference of the ‘gap’ signal, while robust linear regression examined the effect of either age at surgery or follow‐up time on the post–pre difference of the ‘gap’ signal.

**Results:**

The primary finding was a significant reduction in the ‘gap’ signal [−9.2 mm (95% CI: −12.3; −6.7)] between 2 weeks and ~8 months (*p* < 0.001). The ‘normal tendon’ signal did not change significantly [1.6 mm (95% CI: −0.5; 3.3), *p* = 0.237]. Age at surgery had a negative, statistically nonsignificant effect on donor‐site ‘gap’ signal difference (−2.24 [95% CI: −5.59; 0.60], *p* = 0.106). In addition, follow‐up time had a negative, statistically nonsignificant effect on donor‐site ‘gap’ signal difference (−0.97 [95% CI: −2.98; 4.14], *p* = 0.571). Eight of 22 patients had no detectable ‘gap’ signal at 8 months.

**Conclusion:**

The most important finding for this study is a significant decrease in the donor‐site defect along with an undetectable ‘gap’ that was verified for ~36% of the sample at a minimum mean follow‐up of ~8 months. The laterally remaining ‘normal tendon’ signal remained unchanged. Neither age at surgery nor follow‐up time had an impact on the healing of the donor‐site defect. From a clinical perspective our findings suggest that the medial‐third patellar tendon autograft has a high healing capacity after harvesting, already at a mean follow‐up of ~8 months.

**Level of Evidence:**

Level IV.

**Abbreviations**
ACL: anterior cruciate ligament
ACLR: anterior cruciate ligament reconstruction
BPTB: bone–patellar tendon–bone
medial third ACLR: ACLR using the medial third of the patellar tendon
mid third ACLR: ACLR using the middle third of the patellar tendon
MRI: magnetic resonance imaging
‘gap signal’: donor‐site defect size
‘normal tendon’ signal: laterally remaining tendon size

## INTRODUCTION

A bone–patellar tendon–bone (BPTB) autograft is considered the first choice for anterior cruciate ligament (ACL) reconstruction, particularly in young, athletic patients [30]. However, graft selection involves a multifaceted decision influenced by several factors, including donor site morbidity, functional deficits, patient reported outcomes, re‐rupture or infection rates [1, 2, 5, 13, 26, 28, 29]. The BPTB graft is well‐researched and offers several advantages, including its strength and stiffness, which closely match those of the native ACL [26, 33].

A primary concern with using the BPTB graft is postoperative donor‐site morbidity, which may arise due to a variety of factors, such as the patient's characteristics, the surgical graft‐harvesting technique, the postoperative rehabilitation protocol, and the restoration of knee motion. Modern imaging techniques, particularly magnetic resonance imaging (MRI), provide valuable insights into the healing process of the donor‐site defect and the condition of the remaining patellar tendon [11, 16, 27]. Understanding how the defect size changes over time is crucial, as it can have implications for the patient's long‐term recovery, function, and risk of complications.

Previous studies have reported that the ‘restoration’ of the donor‐site defect with normal ‘tendinous‐like’ tissue is rare, even 2‐years after ACL reconstruction [15]. However, this finding contradicts earlier studies that suggested some degree of healing over time [25]. Moreover, healing of the donor‐site defect appears to continue well beyond 6 months postoperatively, although the rate of healing slows after this point [3], with some studies suggesting it may practically cease by 18 months [7]. In contrast, other studies have shown that the defect size continues to decrease gradually up to 27 months postoperatively [15, 19, 32]. Some authors also suggest that healing is less likely if the peritendon is not closed during surgery [31].

While the majority of these studies have examined the time course of donor‐site defects following ACL reconstruction using the middle third of the BPTB autograft (mid‐third ACLR), there is limited data regarding the donor‐site defect following harvesting of the medial third of the BPTB (medial third ACLR) [4, 8, 20]. It has been postulated that the medial third of the patella may offer some advantages in terms of reducing donor‐site morbidity, particularly with respect to avoiding incisions directly over the tibial tubercle [9, 12, 23]. However, understanding the impact of harvesting the medial third of the BPTB on the donor‐site defect is crucial for several reasons. The donor‐site defect may contribute to anterior knee pain, functional deficits, and even long‐term complications such as patellofemoral instability or weakness in the extensor mechanism. In addition, understanding the time course of donor‐site defect healing could have significant implications for revision surgeries. If a substantial defect persists, it may affect the ability to reuse the graft site in the event of a revision ACLR.

Therefore, the aim of the present study was to assess the size of the donor‐site defect following medial third ACLR using serial MRI evaluations. The primary aim was to evaluate how the donor‐site defect changes over time and to determine if a significant decrease occurs within the first year. Our hypothesis was that the donor‐site defect would significantly decrease at least 6 months after the index procedure.

## PATIENTS AND METHODS

This was a prospective observational study and explicit patient selection criteria were established to maintain the homogeneity of the population sample and ensure the validity of the results. Therefore, for the present study, male patients who had undergone medial third patellar tendon ACLR were enroled. Patients with more than 25% of meniscus damage, multi‐ligament injuries, revision operation of the ACL ligament, as well as serious chondral lesions (Outerbridge classification III or IV), were excluded from this study. Finally, because of socioeconomic reasons, patients living more than 1 h away from the hospital where the present study was conducted were also excluded. All patients that agreed to undergo serial MRI evaluations were finally included in the study. All patients had an uninjured contralateral knee. Informed consent was obtained from all patients enroled in this study, in accordance with the institutional review board policies of the local University Hospital.

### Surgical procedure

**Figure 1:**
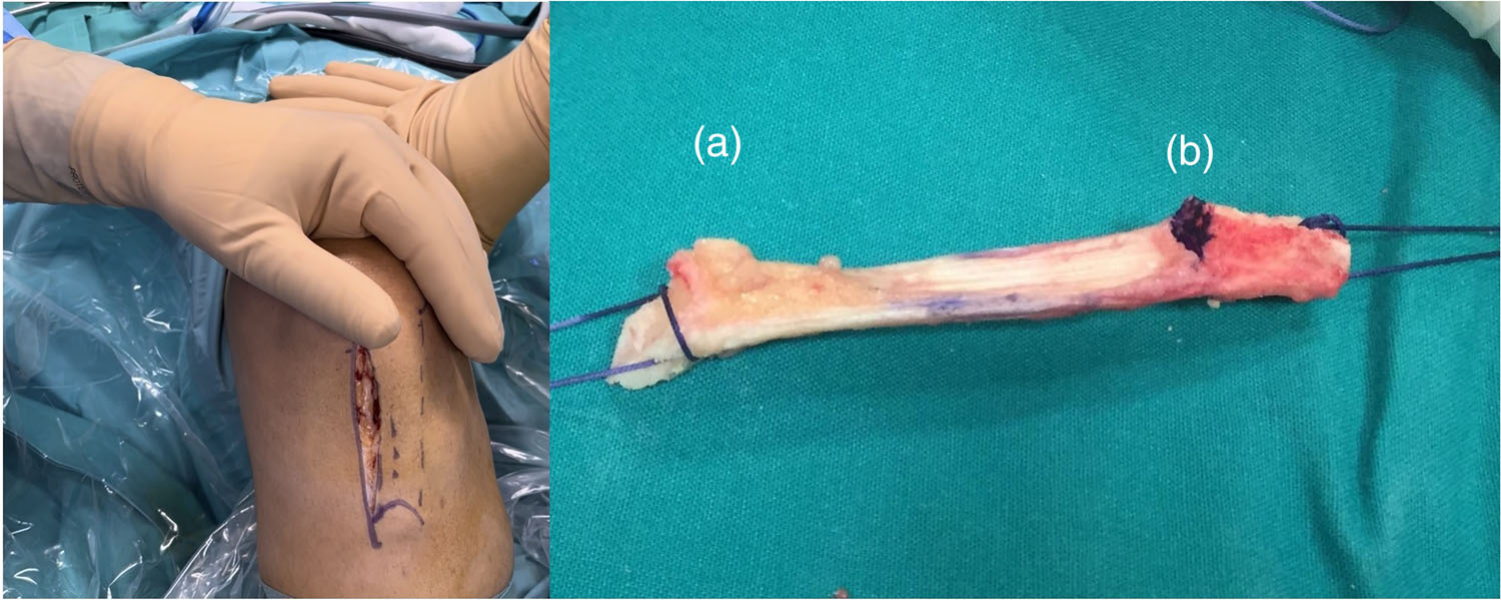
Harvesting site of the medial third of the patellar tendon (left knee). (a) Vertical medial incision, extending from the inferior medial edge of the patella to the medial side of the tibial tuberosity. (b) Medial third of the patellar tendon and the accompanying bone pieces (a, inferior surface of the patella, b, tibial tuberosity).

The graft was harvested from the medial third of the patellar tendon [8]. A vertical medial incision of approximately 6–8 cm in length extended from the inferior medial edge of the patella to the medial side of the tibial tuberosity (Figure 1a). After the subcutaneous tissues were dissected, the patellar tendon was fully exposed. The autograft included the medial third of the patellar tendon accompanied by two bone pieces: one from the inferior surface of the patella (10 × 10 mm) and one from the tibial tuberosity (20 × 25 mm) (Figure 1b). The graft was harvested using an osteotome and precision saw, aiming for a 10 mm diameter. Particular care taken to avoid fractures, especially of the patella. Due to small size, the patellar defect was not grafted; however the tibial defect was grafted. The preparation of the graft included shaping the bone pieces to precisely fit the bone tunnels that would subsequently be created in the femur and tibia. Two holes were drilled at each bone end to pass sutures. The graft was kept in a solution of saline with vancomycin until its implantation. The femoral bone tunnel was created arthroscopically through the anteromedial portal with the knee in 120° flexion, and the tunnel was positioned at the center of the anatomical ACL insertion. The tibial bone tunnel was positioned 2 mm posterior to the center of the native ACL insertion. The graft was positioned in the femoral tunnel with the cortical bone of the tibial graft facing the roof of the intercondylar notch (over‐the‐top position). The graft was fixed with bioabsorbable screws in both the femur and tibia, ensuring the proper tension and anatomical alignment of the graft. The final fixation in the tibia was performed with the knee at 30° flexion while maintaining graft tension. A final check was performed throughout the full range of motion to ensure no impingement on the intercondylar roof, lateral femoral condyle, or posterior cruciate ligament [9].

### Clinical evaluation

All patients followed the same carefully controlled rehabilitation protocol. Clinical examination was performed by an independent experienced orthopaedic surgeon at the same day that the MRI examinations and included clinical evaluation of knee joint stability (Lachman, anterior drawer, and pivot‐shift tests).

### MRI evaluation

Each patient underwent static MRI of the knee joint. For all patients the MRI examinations involved the donor‐site defect (‘gap’ signal) as well as of the laterally remaining tendon (‘normal tendon’ signal) on both occasions. All examinations were performed on the same 1.5‐T MRI unit (Gyroscan ACS NT; Philips, Medical System Best) by using a circular receiver–transmission extremity coil (field of view 180 mm and acquisition matrix 256 × 256). The knee was positioned in full extension, at a slight external rotation (10–15°). The standard examination protocol included a series of sequences in orthogonal planes, in order to obtain an overview of the patellar tendon and joint structures: axial turbo spin‐echo proton density‐weighted sequence with fat saturation (TR/TE 3450/15 ms, slice thickness/intersection gap 4.00/0.4 mm), coronal and sagittal turbo spin‐echo proton density weighted sequences (TR/TE 2750/16 ms, slice thickness/intersection gap 3.00/0.3 mm), coronal short Tau inversion recovery (TR/TI/TE 1780/160/55 ms, slice thickness/intersection gap 3.00/0.3 mm), sagittal turbo spin‐echo T2‐weighted sequence with fat suppression (TR/TE 2600/70 ms, slice thickness/gap 3.00/0.3 mm) and sagittal three dimensional (3D) fast field echo sequence with fat suppression (TR/TE 32/5.1 ms, slice thickness 2.0 mm).

All measurements were performed on the axial turbo spin‐echo proton density‐weighted sequence with fat saturation (TR/TE 3450/15 ms, slice thickness/intersection gap 4.00/0.4 mm) at the level of the lower 1/3 of the patellar tendon. Measurements were made on the transverse level and on the same slice: (1) latero‐lateral diameter concerning the medial part of the patellar tendon that had been removed and appeared with a profoundly increased signal on the above sequence (‘gap’ signal) and 2) latero‐lateral diameter of the laterally remaining tendon that displayed a profound low signal on the above sequence (‘normal tendon’ signal).

The intra‐observer standard deviation of the difference between two measurements was 1.3 mm as assessed by reevaluating 15 randomly selected examinations of normal patellar tendons without knowledge of the primary result. The corresponding intra‐class correlation coefficient was 0.98 (95% CI: 0.94, 0.99) indicating excellent reliability.

### Statistical analysis

Values are reported as mean (±95% confidence intervals) unless otherwise stated. Wilcoxon signed rank two samples test was used to compare MRI signal between the two time‐point measurements (Delta_post‐pre_) for the donor‐site ‘gap’ signal and the lateral remaining ‘normal tendon’ signal against the *H*_0_ (Delta_post‐pre_ = 0). Robust linear regression was used to evaluate the effect of either age at surgery or follow‐up time on donor‐site ‘gap’ signal Deltapost‐pre [18]. This method relies on rank‐based estimation and is insensitive to outliers and violations of normality. To facilitate the interpretation of model‐estimated regression coefficients (slopes), age at surgery and follow‐up time were expressed as *z*‐scores. To evaluate the stability and uncertainty of these slopes, confidence intervals were derived using nonparametric bootstrap resampling methods (*n* = 1000) [6], which do not rely on distributional assumptions and are suitable for robust estimation frameworks. An effect was considered statistically significant if the confidence interval did not include zero. All analyses were performed in the environment of the open programming language R (v.4.3.2). The level of significance was set at *a* = 0.05.

## RESULTS

**Figure 2:**
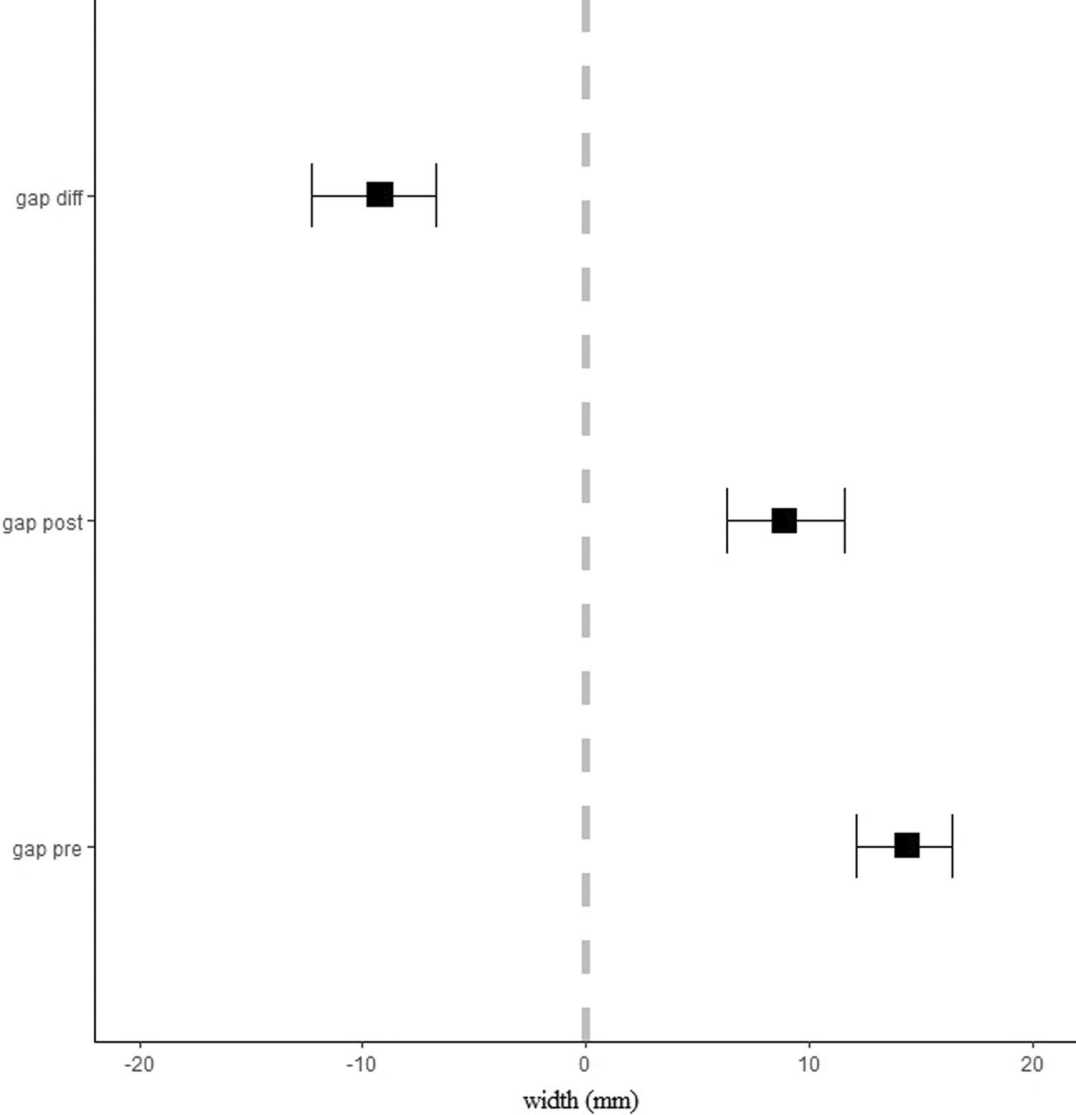
Size of the ‘gap’ signal at the pre‐ and post‐MRI evaluation as well as the post–pre difference. Values are mean ± 95% confidence intervals.

**Figure 3:**
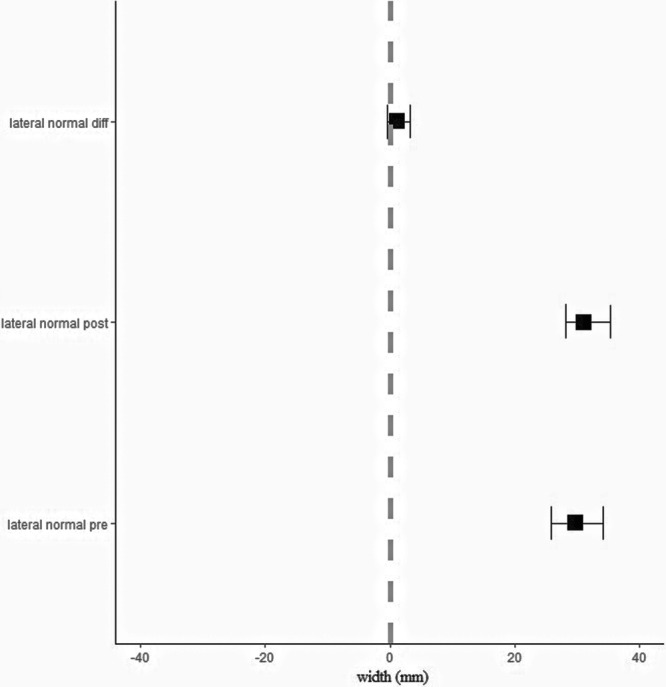
Size of the ‘normal tendon’ signal at the pre‐ and post‐MRI evaluation as well as the post–pre difference. Values are mean ± 95% confidence intervals.

**Figure 4:**
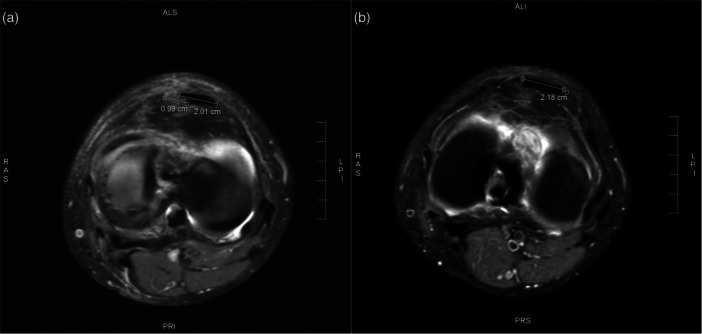
Serial axial image of the operated knee 3 weeks postoperatively with both a profound increased signal at the harvested medial part of the patellar tendon (‘gap’) and a profound low signal (laterally remaining ‘normal patellar tendon’) (a). Serial axial image of the operated knee at 5.4 months postoperatively (b). Note that there is no measurable ‘gap’ signal, yet there is still a low signal (laterally remaining ‘normal patellar tendon’).

Patients at the time of study enrolment were 25.7 ± 6.4 years old and performed the MRI evaluations at 14 (±5) days and 7.9 (±2.0) months postoperatively. The size of the ‘gap’ signal decreased significantly [Deltapost‐pre = −9.2 mm (95% CI: −12.3; −6.7), *p* < 0.001] between 2 weeks and ~8 months (Figure 2). The size of the ‘normal tendon’ signal did not change significantly [Deltapost‐pre = 1.2 mm (95% CI: −0.5; 3.3), *p* = 0.237] (Figure 3). Age at surgery had a negative, statistically nonsignificant effect on donor‐site ‘gap’ signal difference (−2.24 [95% CI: −5.59; 0.60], *p* = 0.106). In addition, follow‐up time had a negative, statistically nonsignificant effect on donor‐site ‘gap’ signal difference (−0.97 [95% CI: −2.98; 4.14], *p* = 0.571). In eight out of the 22 patients there was no detectable ‘gap’ signal in the post evaluation (Figure 4). There were no differences between these patients and the rest of the sample in terms of follow‐up time, age at surgery and clinical evaluation of knee joint stability.

## DISCUSSION

The aim of the present study was to report the healing time course of the donor‐site defect at short‐term follow‐up after medial‐third patellar tendon ACLR. The most important finding for this study is that at a minimum follow‐up of ~8 months, the defect size of the donor site had healed by −9.2 mm (95% CI: −12.3; −6.7) leaving a mean of 5.3 ± 5.5 mm defect width according to MRI measurements of the donor site. Furthermore, during the same observation period the size of the laterally remaining ‘normal tendon’ signal did not change significantly. No measurable ‘gap’ signal was verified for 8/22 patients. There was no statistically significant effect of either age at surgery or follow‐up time on the healing of the donor‐site defect. Collectively, our findings suggest that the medial‐third patellar tendon autograft has a high healing capacity after harvesting, so that at a mean follow‐up of ~8 months, 36.4% of the sample had reached full defect closure.

Several studies have examined the healing process of the donor‐site defect following mid‐third patellar tendon ACLR [3, 6, 7, 15, 17, 19, 25, 31, 32]. To the best of the author's knowledge, the healing of the donor‐site defect following medial‐third patellar tendon ACLR has only been investigated in early animal models [4, 20], and therefore this study could serve as baseline evidence, given that the healing process is less known compared to mid‐third patellar tendon authografts. For example, Bernicker et al. [3] reported a reduction from an initial mean value of 10.9 mm at 3 weeks postoperatively to 9.4 mm at 3‐months postoperatively and 5.9 mm at 6‐months postoperatively [3]. These authors demonstrated a 62% decrease of the patellar tendon defect at 12‐months after the index operation [3]. Similar findings have been reported by Kartus et al. [15] [9 mm (4–18) at 6 weeks postoperatively to 5 mm (2–14) at 6 months postoperatively]. More recently, Svensson et al. [32] reported gap signal size decrease from 8.4 ± 3.6 mm at 6 weeks postoperatively to 4.7 ± 2.7 mm at 6 months postoperatively, 2.0 ± 1.4 mm at 27 months and 0.5 ± 0.9 at 71 months postoperatively.

Regarding the proportion of patients with no ‘detectable’ gap signal, there is quite large variation between studies. For example, Kartus et al. [15] reported no ‘detectable’ gap signal for only 1 of 31 patients during the 2‐year follow‐up. In addition, these authors reported that the majority of the patients still had a palpatory defect in the patellar tendon. Bernicker et al. [3] reported that 5/12 patients had formed no soft tissue over the patellar harvest site at the 6 months evaluation, while 7 had a ‘thin layer’ continuous with the adjacent pre‐patellar expansion (but <50% of its normal thickness). At the 12 months evaluation there were 3 patients that still had no significant soft tissue formation, whilst 5 had a “thin layer”. The authors report that 4/12 patients had donor‐site defect healing at the 12‐months evaluation. More recently, a comparative study of patients with short (≤12‐months, *n* = 14) and long (>12‐months, *n* = 14) postoperative intervals showed complete absence of gap signal only 1 out of 14 patients (7%) for the short postoperative group [19]. On the contrary, complete absence of gap signal at the donor site was seen in the 6 out of 14 patients (42%) for the long postoperative group. The authors concluded that up to 1 year after mid‐third patellar tendon ACLR there is no complete elimination of the gap signal at the donor site. Similarly, Svenson et al. [32] reported that absence of ‘gap’ signal at the donor‐site defect was detected for 3 out of 19 patients (along with tendinous‐like tissue signal) [32] 2 years postoperatively. The corresponding proportion of nondetectable gap signal was 13 out of 17 patients at 6‐years. It appears that, for most studies, detection of patients with complete elimination of the gap signal at the donor defect site requires follow‐up periods of more than 12‐months. Our study has comparable proportions of nondetectable gap signal with these studies [3, 15, 19, 32]; however, our follow‐up period is quite shorter.

From a clinical perspective the time course of the donor‐site defect healing is of special interest in the case of BPTB ACLR given that previous studies have attributed donor‐site morbidity, specifically anterior knee pain, to the graft harvest process [12, 13, 18]. Studies have shown a wide range of postoperative donor site morbidity with a BPTB graft, from as low as 8% to more than 50% of patients with subjective complaints [13, 15, 17, 26]. However, it appears that along with graft harvest process, intraoperative procedures can also have an impact on donor‐site morbidity/adverse effects [10, 12, 21, 22, 24]. In addition, although BPTB ACLR can alter the position of the patella, harvesting of the medial‐third of the patellar tendon is performed without damage to the attachment of the medial patella‐femoral ligament, the patella‐meniscal ligament, or the medial retinacular fibres, thus maintaining them provides restraining mechanisms that prevent lateral displacement of the patella [14]. Furthermore Moebius et al. [23] concluded that alterations of the extensor apparatus after medial‐third patellar tendon ACLR were similar to those after mid‐third ACLR. Finally, medial‐third patellar tendon ACLR avoids direct incision over the tibial tubercle [9] and may be associated with lesser direct incisional pressure during activities such as kneeling [12].

Potential limitations of the present study, is the lack of a control group because of logistical and ethical reasons to perform serial MRI examinations on subjects without known knee pathology. The sample appears small; given the strict eligibility criteria we consider it as fair. In addition, MRI evaluates the appearance but not the underlying composition of the donor site defect; the latter would necessitate the need for biopsies and histological analyses. In addition, the clinical relevance between MRI donor‐site defect closure findings and patient outcomes remains theoretical; however, our results on the rapid resolution of the gap signal imply efficient regeneration of the tissue and potentially minimal impact to the extensor mechanism.

## CONCLUSIONS

The most important finding for this study is a significant decrease in the donor‐site defect along with an undetectable ‘gap’ that was verified for ~36% of the sample at a minimum mean follow‐up of ~8 months. The laterally remaining ‘normal tendon’ signal remained unchanged. Neither age at surgery nor follow‐up had an impact on the healing of the donor‐site defect. From a clinical perspective our findings suggest that the medial‐third patellar tendon autograft has a high healing capacity after harvesting, already at a mean follow‐up of ~8 months.

## AUTHOR CONTRIBUTIONS

Jim Georgoulis designed the search strategy, conducted the search, screened the papers, performed the statistical analysis extracted the data and wrote the manuscript. Olga Savvidou assisted in designing the search strategy. Paraskevi Kosta assisted in conducting the search. Kostas Patras assisted in the statistical analysis. Maria Argyropoulou and Panayiotis Papagelopoulos reviewed the findings of the analysis and contributed to the discussion. Anastasios Georgoulis assisted in designing the search strategy, reviewed the findings of the analysis, verified scientific merit, and contributed to the discussion. All authors reviewed and approved the final manuscript.

## CONFLICT OF INTEREST STATEMENT

The authors declare no conflicts of interest.

## ETHICS STATEMENT

Attikon University General Hospital Scientific Board, ΑΡΘΟΠ, ΕΒΔ 3/18.01‐2021. All patients provided informed consent.

## Data Availability

The data that support the findings of this study are available from the corresponding author upon reasonable request.
